# Ascertaining Household-Level Exposure to Air Pollution: Socio-Demographic Patterning and Urban-Rural Differences in People with Schizophrenia and Other Psychotic Disorders and the General Population

**DOI:** 10.23889/ijpds.v11i1.3360

**Published:** 2026-07-02

**Authors:** Ella S. Christoforou, Jane Lyons, Ann John, Rich Fry, Martin J.D. Clift, Amy Mizen

**Affiliations:** 1 Institute of Life Science, Swansea University Medical School, Swansea SA2 8PP, UK; 2 Population Data Science, Swansea University Medical School, Swansea SA2 8PP, UK; 3 In Vitro Toxicology Group, Institute of Life Science, Swansea University Medical School, Swansea SA2 8PP, UK

**Keywords:** schizophrenia, psychotic disorders, air pollution, deprivation, electronic health records

## Abstract

**Background:**

People with severe mental disorders, including schizophrenia, experience worse physical health and shorter life expectancy. While socio-demographic factors are well established contributors to these disparities, less is known about how environmental exposures differ between individuals with severe mental disorders and the general population.

**Objective:**

To explore socio-demographic and environmental differences between the general population and individuals with schizophrenia and/or other psychotic disorders (OPD).

**Methods:**

Anonymised general practice records (2016–2019) for individuals resident in Wales were accessed within the Secure Anonymised Information Linkage (SAIL) Databank. This anonymised health data were linked to small-area level air pollution data for 2016 (PM_10_, PM_2.5_, NO_x_) for individuals who remained at the same residential address during the study period. Individuals with a first relevant diagnosis were identified; age, sex, urban/rural residence, deprivation, and air pollution exposures were compared with the general population.

**Results:**

Overall, 0.1% (1,784) of the SAIL population had a first recorded diagnosis of schizophrenia/OPD. Of these, 52.7% (941) were male and 47.3% (843) were female. The psychiatric cohort had a higher mean age at study entry than the general population (46.9 vs 42.3 years). The distribution of diagnoses in urban (72.3%) and rural (27.7%) areas closely matched the total population (urban 70.5%, rural 29.5%). Mean air pollutant levels (PM_10_, PM_2.5_, NO_x_) in 2016 were similar between the psychiatric cohort and the general population. A clear socioeconomic gradient was observed: 29.1% of cases resided in the most deprived quintile compared with 15.0% in the least deprived quintile.

**Conclusions:**

Area-level deprivation was associated with higher prevalence of schizophrenia/OPD, whereas no clear differences were observed by urbanicity or air pollution exposure. Linked population-scale health and environmental data provide valuable evidence which could inform service planning and targeted public health interventions.

## Introduction

Mental health problems can affect anyone and have a significant effect on the lives of individuals, their families, communities, and wider society. Globally, one in eight people live with a mental disorder [1] and the rates of mental illness have been gradually rising [2, 3]. In the UK, approximately 4.6 million people were diagnosed with a mental disorder (excluding alcohol and drug use disorders) in 2019 [4]. This equates to approximately one in four adults experiencing at least one diagnosable mental health condition annually [5]. In Wales, estimates suggest that severe mental illness (SMI) affects around 2% of the population, equivalent to approximately 62,150 people [6]. Within this group, schizophrenia and other psychotic disorders (OPD) represent a substantial proportion and are associated with significant functional impairment and long-term health inequalities.

Mental illness is closely associated with many forms of inequality. Health inequalities are avoidable and unfair differences in health status between groups of people. These differences arise from variations in social and economic determinants such as demographic, socioeconomic, and geographical factors [7]. Patients suffering from severe mental disorders, such as schizophrenia, have a reduced life expectancy (up to 10–25 years) compared to the general population [8]. Evidence also suggests that the mortality gap is widening [7]. The high mortality rate is a consequence of the simultaneous presence of comorbid physical health problems, such as cardiovascular, respiratory, and infectious diseases in addition to cancer [9, 10].

Environmental and socioeconomic factors may contribute to these inequalities. Air pollution represents a major global health challenge and is often more pronounced in urban environments and in lower income countries [11]. In the most deprived areas there are often higher air pollution levels [12], increased risk of mental illness [13] and decreased life expectancy compared to less deprived areas [14]. Furthermore, the risk of mental disorders is generally higher in more urbanised areas compared to less urbanised or rural areas [15–17]. However, a review [18] revealed complex patterns of urbanicity–psychosis associations with international variation within Europe and between low, middle, and high-income countries. The observed heterogeneity is likely due to multiple interacting risk factors e.g., air pollution and protective factors (e.g., greenness) [18, 19].

Growing epidemiological evidence suggests that exposure to air pollution may contribute to the onset and exacerbation of severe mental disorders, including schizophrenia [20]. Proposed biological mechanisms include neuroinflammation, oxidative stress, altered gene expression, and structural brain changes [20]. Empirical findings support this association. A national cohort study in Denmark found that childhood exposure to nitrogen oxides (NO_x_) was associated with an increased risk of schizophrenia after adjustment for deprivation and urbanicity [21]. However, findings were less consistent for particulate matter of different fractions (PM_10_ and PM_2.5_) [21]. Similarly, a Danish population-based cohort study demonstrated that higher childhood nitrogen dioxide (NO_2_) exposure and polygenic risk scores were independently associated with increased schizophrenia risk [22]. However, these studies did not describe or compare the environmental contexts of individuals with and without schizophrenia. Using UK Biobank data, long-term exposure to multiple air pollutants (PM_2.5_, PM_10_, NO_x_, and NO_2_) in adulthood has been associated with schizophrenia risk across genetic risk groups [23]. An analysis was also conducted describing urbanicity, deprivation, age, and sex in schizophrenia and non-schizophrenia populations.

Hence, existing studies have largely focused on estimating associations rather than describing and comparing the broader socio-demographic and environmental contexts of individuals with and without psychotic disorders. Moreover, evidence derived from routinely collected, population-level data remains limited [23].

This study builds on existing evidence by using linked, routinely collected population-level data from Wales. The linked data were used to describe and compare socio-demographic and environmental exposures, including deprivation, urbanicity, and air pollution, between individuals with psychotic disorders and the general population. This population-wide descriptive analysis establishes a baseline of inequalities to guide future research, service planning, and policy development [7].

**Aim:** To describe the socio-demographic and environmental differences between the general population and those with schizophrenia/OPD.

Hypothesis: Individuals diagnosed with schizophrenia/OPD are more likely to reside in areas with higher air pollution levels and greater deprivation compared to the general population.

**Objectives:**

1. Create a longitudinal cohort of individuals living in Wales from 2016-2019 and flag individuals with their first diagnosis of schizophrenia/OPD during this time.
2. Link air pollution data with demographic and health data.
3. Calculate descriptive statistics e.g., age, sex, deprivation level, urban/rural classification, and air pollutants level in the general population and in those with schizophrenia/OPD.

## Methods

### Data Linkage

Previous annual air pollution data from 2016 at the Lower Layer Super Output Area (LSOA) level was linked to individuals registered with a Welsh General Practice (GP) between 1 July 2016 to 30 June 2019. Anonymised, longitudinal information on health, social and environmental data on the Welsh population is contained in the Secure Anonymised Information Linkage (SAIL) Databank [24, 25]. The SAIL databank allows consistent data linkages at the individual and household level. The study population comprised approximately 2 million people living in Wales who were registered with SAIL-contributing GP practices. Eligible participants were those living in Wales and registered with a Welsh GP throughout the full 3-year follow-up period (1 July 2016 to 30 June 2019).

**Figure 1:**
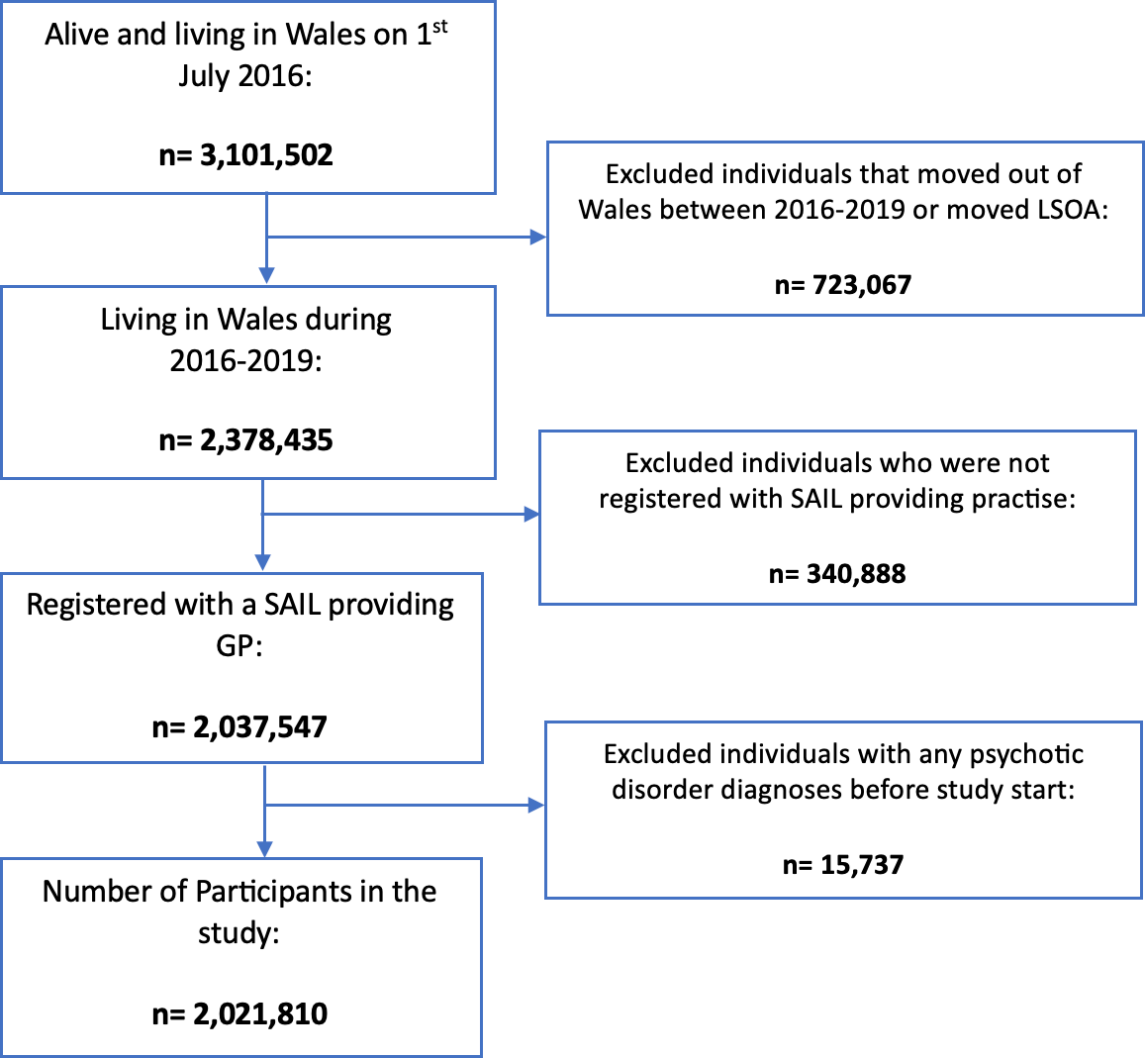
Flow Diagram Illustrating Cohort Derivation and Participant Selection

The psychiatric cohort included individuals with their first schizophrenia/OPD diagnosis during this period. This timeframe was selected to avoid the COVID-19 pandemic, during which GP services were disrupted and patients reported difficulty accessing treatment [26]. To ensure accurate assignment of 2016 air pollution, participants were required to have a stable residential address throughout the study period. No restrictions were applied regarding age, sex, or environmental characteristics. Figure 1 illustrates the derivation of the final cohort.

### Study Area and Subjects

The study was conducted in Wales, a nation of approximately 3.13 million [27]. The population is predominantly concentrated in the south of the country, particularly in the urban centres of Cardiff (∼372,000 residents) and Swansea (∼241,000 residents) [27]. Air pollution levels vary across Wales. In 2020, South Wales recorded the second-highest annual mean NO_2_ concentration in the UK (64 *μ*g/m^3^), after London (77 *μ*g/m^3^), exceeding the UK and EU legal limit of 40 *μ*g/m^3^ [28, 29]. The North Wales zone and Cardiff Urban Area also exceeded the national legal limit (with 44 and 42 *μ*g/m^3^ respectively) [28]. Annual mean PM_2.5_ concentrations in four out of ten Welsh locations exceeded the recommended WHO guidelines of 10 *μ*g/m^3^ [30, 31]. Overall, 88% of the Welsh population resides in built-up areas [32].

### Data Sources

#### Mental Health Data

Schizophrenia/OPD were identified using the Welsh Longitudinal General Practice (WLGP) dataset. This dataset contains individual-level primary care records, including Read codes documenting diagnoses, symptoms and treatments for each patient. Read codes are a standardised clinical coding system used within the NHS since 1985 to record patient information across primary and secondary care settings [33]. Code lists were obtained from the SAIL Databank Concept Library for schizophrenia-related disorders (schizophrenia, schizotypal and delusional disorders) [34]. In addition to code lists for other psychotic disorders such as acute and transient psychotic disorders, depressive episodes/disorders with psychotic symptoms [35]. To identify incident cases, individuals with a recorded diagnosis of schizophrenia or other psychotic disorder prior to the study start date (1 July 2016) were excluded.

#### Air Pollution Data

This study was designed as a pilot analysis using the SAIL Databank to assess the feasibility of linking area-level air pollution with mental health outcomes. Data from a single year were used to provide a snapshot of exposure while limiting analytical complexity in this initial investigation. Although multi-year modelled air pollution data are available via the Department for Environment, Food & Rural Affairs (Defra), using multiple years of data was beyond the scope of this pilot study and is being addressed in ongoing work.

Air pollution data were obtained from DataMapWales [36], an open-access platform developed through a partnership between the Welsh Government and Natural Resources Wales. The data are derived from the UK-wide Defra air pollution dataset. For this study, annual mean concentrations of PM_10_, PM_2.5_, and NO_x_ for 2016 were aggregated from 1 km^2^ grid resolution to the LSOA level. Air pollution values were assigned from the 1km grid where the LSOA centroid was located using QGIS version 3.3. LSOAs are the smallest units of geography at which census data is estimated [37]. There are 1,909 LSOAs in Wales, each containing approximately 400-1,200 households and 1,000 and 3,000 residents [37], with a mean population of approximately 1,500 [38]. LSOAs are designed to be as homogenous as possible in terms of type of residence and urban/rural areas [37]. The 2011 rural-urban classification of LSOAs was obtained from the Office for National Statistics (ONS) [39].

#### Demographic and Socioeconomic Data

To assign air pollution exposure and classify urban/rural status the Welsh Demographic Service dataset (WDS) was used. The WDS is a population register that contains administrative information and anonymised historical home addresses of patients registered with a Welsh GP.

The residential linkages in this dataset allow accurate allocation of individual-level air pollution exposure, creating high spatial resolution data [40]. Furthermore, this also enables inclusion of individuals who continuously resided within the same LSOA during the study period (1^st^ July 2016 to 30^th^ June 2019). Additionally, the WDS provides key demographic information, including age and sex.

Deprivation at the LSOA level was derived from the Welsh Index of Multiple Deprivation (WIMD) dataset which is the Welsh Government’s official measure from 2019 [41]. WIMD identifies areas with the highest concentrations of eight different types of deprivation: income, employment, health, education, housing, access to services, community safety and physical environment [42]. LSOAs are ranked from 1 (most deprived) to 1,909 (least deprived) [41]. An area has a higher deprivation rank than another if the proportion of people living there are classified as deprived is higher. WIMD is a National Statistic produced by statisticians at the Welsh Government. A summary of the data sources and their coverage are presented in Table 1.

**Table 1:** Summary of Datasets Used in this Study, Including Data Sources, Derived variables, and Population or Geographic Coverage

Dataset name	Data source	Derived variables	Coverage
Air pollution	DataMapWales (derived from [Defra])	Annual mean concentrations of PM_10_, PM_2.5_ and NO_x_ (2016)	For each LSOA in Wales (n=1,909).
Welsh Longitudinal General Practice (WLGP)	Primary care records.	Diagnoses of schizophrenia/OPD (Read codes)	∼80% of GP practices in Wales.
Welsh Demographic Service (WDS)	Digital Health and Care Wales	Age, sex, week of birth, residential history.	All individuals registered with a GP in Wales.
Welsh Index of Multiple Deprivation (WIMD)	Welsh Government.	Area-level deprivation quintile (1 = most deprived; 5 = least deprived)	LSOA level (2019)
Rural/urban Classification	Office for National Statistics.	Urban or rural.	All LSOAs in Wales (n = 1,909)

### Statistical Analysis

All analyses were conducted in R version 4.1.3 within the SAIL Databank. The dplyr package was used to generate summary statistics. Descriptive statistics were reported for the study population and for individuals with schizophrenia/OPD). The descriptive statistics included: sex (male/female), age at study start (mean, median, interquartile range [IQR]), concentrations of NO_x_, PM_10_ and PM_2.5_ (mean, minimum, median, maximum), rural urban classification and deprivation level. The R code used to generate these summary statistics is provided in the supplementary materials.

## Results

Using a combination of open-source environmental and national mapping agency data, the feasibility of creating individual-level longitudinal environmental exposure data across Wales was demonstrated. In total, 2,021,810 individuals were included in the analysis. These individuals were registered with a Welsh GP practice contributing data to the SAIL Databank and remained at the same residential address between the 1 July 2016 to 30 June 2019. Within this population, 0.1% (1,784) had a diagnosis of schizophrenia/OPD. The number and percentage of individuals with recorded diagnoses of schizophrenia/OPD within the study population is presented in Table 2.

**Table 2:** Number and Percentage of Individuals in the Study Population with Recorded Diagnoses of Schizophrenia/ OPD in Welsh Primary Care Records

		**Percentage of**
**Participants**	**Number**	**all participants (%)**
Total population	2,021,810	(100.000)
Schizophrenia only	899	(0.044)
OPD only	776	(0.038)
Schizophrenia & OPD	109	(0.005)
Schizophrenia or OPD	1,784	(0.088)

**Figure 2:**
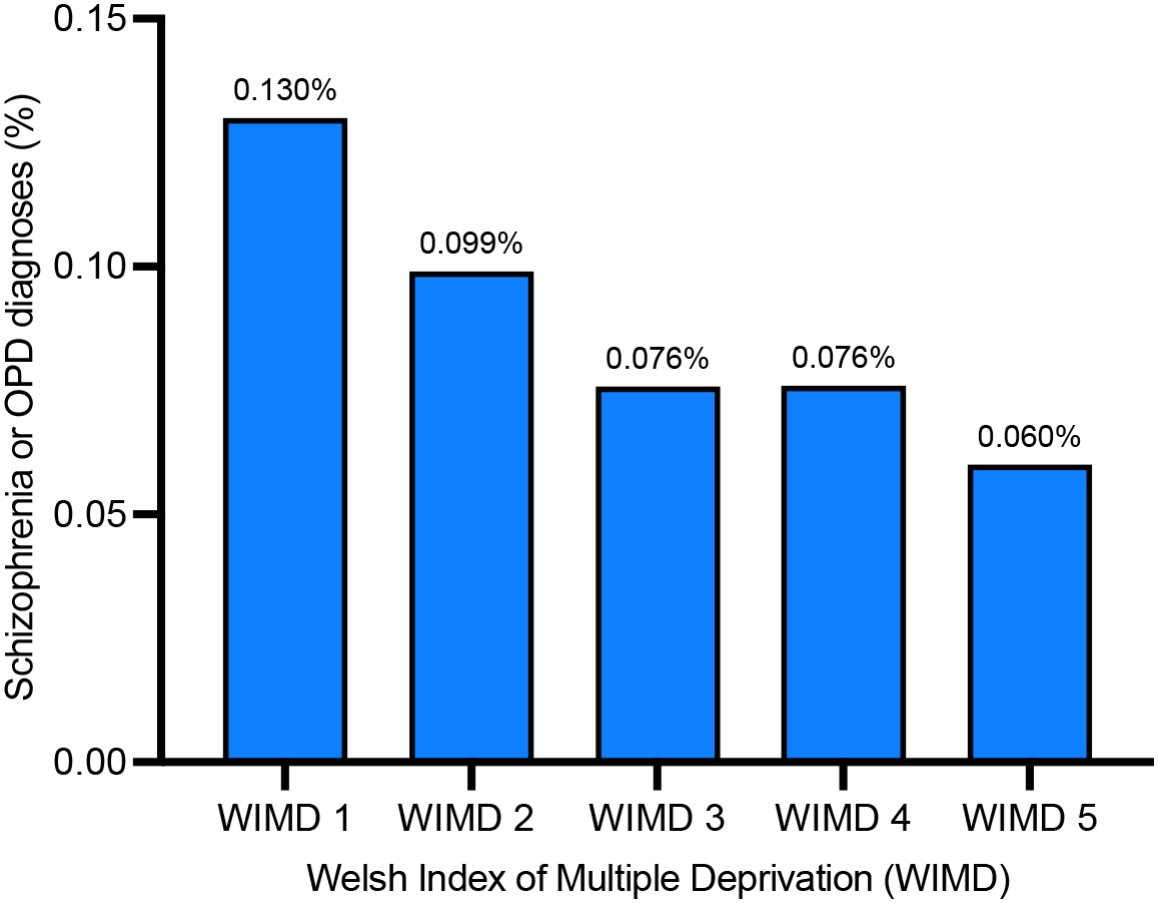
Distribution of Schizophrenia/OPD Diagnoses Across Welsh Index of Multiple Deprivation Quintiles

The total cohort had a nearly equal distribution of males (50.2%) and females (49.8%). Among the 1,784 individuals who developed schizophrenia/OPD, a slightly higher proportion were male (52.7%) compared to female (47.3%). The mean age of the total cohort was 42.3 years (median 44, IQR 23–61), whereas individuals with incident schizophrenia/OPD were older on average, with a mean age of 46.9 years (median 45, IQR 28–65). Regarding residential environment, 70.5% of the total cohort resided in cities and towns, while 29.5% lived in rural or fringe areas. A similar pattern was observed among incident cases, with 72.3% living in urban areas and 27.7% in rural/fringe areas. Deprivation, measured using the WIMD, showed that the highest incidence of schizophrenia/OPD was observed among individuals in the most deprived quintile (29.1%), whereas the least deprived quintile accounted for 15.0% of incident cases, indicating a social gradient in the distribution of schizophrenia/OPD diagnoses (Figure 2). This information is presented in Table 3.

**Table 3:** Comparison of Demographic, Socioeconomic, and Environmental Characteristics Between the Total Study Population and Individuals with Incident Schizophrenia/OPD

Characteristic	Total cohort (n= 2,021,810)	Incident schizophrenia/OPD (n = 1,784)
**Sex**		
Male	1,014,575 (50.2)	941 (52.7)
Female	1,007,235 (49.8)	843 (47.3)
**Age**		
Mean	42.3	46.9
Median	44	45
IQR	23-61	28-65
**Environment**		
Rural and fringe	596,594 (29.5)	494 (27.7)
City and town	1,425,216 (70.5)	1,290 (72.3)
**WIMD**		
1-Most deprived	413,529 (20.5)	519 (29.1)
2	411,408 (20.3)	408 (22.9)
3	393,140 (19.5)	298 (16.7)
4	384,453 (19.0)	292 (16.3)
5 - Least deprived	419,280 (20.7)	267 (15.0)

Annual mean concentrations of PM_10_, PM_2.5_, and NO_x_ in 2016 for the schizophrenia/OPD cohort and for all participants are presented in Tables 4–6. NO_x_ encompasses nitric oxide (NO) and nitrogen dioxide (NO_2_), representing total nitrogen oxide emissions [43]. WHO guideline values are provided for PM_10_ and PM_2.5_ for contextual comparison, while no guideline exists for NO_x_ as a combined metric. However, guideline values are available for NO_2_ alone.

**Table 4:** Annual Mean Outdoor PM_10_ Concentrations at the LSOA Level for All Participants and Those with Schizophrenia/OPD

**PM_10_ (μg/m^3^)**	**All Participants**	**Participants with Schizophrenia/OPD**
Mean	11.60	11.59
Minimum	7.69	7.69
Median	11.65	11.64
Maximum	16.31	16.31

WHO annual mean guideline values: 20 *μ*g/m^3^ (2005–2020); 15 *μ*g/m^3^ (2021 update).

**Table 5:** Annual Mean Outdoor PM_2.5_ Concentrations at the LSOA Level for All Participants and those With Schizophrenia/OPD

**PM_2.5_ (μg/m^3^)**	**All Participants**	**Participants with Schizophrenia/OPD**
Mean	7.38	7.37
Minimum	4.83	4.83
Median	7.41	7.42
Maximum	10.64	10.64

WHO annual mean guideline values: 10 *μ*g/m^3^ (2005–2020); 5 *μ*g/m^3^ (2021 update).

**Table 6:** Annual Mean Outdoor NO_x_ Concentrations at the LSOA Level for All Participants and Those With Schizophrenia or OPD

**NO_x_ (μg/m^3^)**	**All Participants**	**Participants with Schizophrenia/OPD**
Mean	15.92	15.75
Minimum	3.96	4.02
Median	14.16	14.32
Maximum	54.33	54.33

No WHO air quality guideline value is available for NO_x_.

## Discussion

A cohort of individuals with their first GP diagnosis of schizophrenia/OPD between 2016-2019 was identified, representing 0.1% of the population. Within the psychiatric cohort, 52.7% were male and 47.3% were female, compared with an equal sex distribution (50.2% male; 49.8% female) in the general population. The psychiatric cohort had a higher mean age at study entry (46.9 years) than the general population (42.3 years). Among those with a diagnosis, 72.3% resided in urban areas and 27.7% in rural areas, closely reflecting the distribution of the general population (70.5% urban; 29.5% rural). Mean levels of PM_10_, PM_2.5_, and NO_x_ were similar between the psychiatric cohort and the general population. In contrast, a clear socioeconomic gradient was observed: 29.1% of individuals with schizophrenia/OPD lived in the most deprived quintile compared with 15.0% in the least deprived quintile.

Previous research indicates schizophrenia incidence is higher in men than in women, with an approximate ratio of 1.4:1 [44]. However, this pattern is less consistent when considering lifetime prevalence [45]. Gender differences are also observed in age at onset, with men typically diagnosed between 15 and 24 years and women more commonly after age 40 [45–47].

A population-based linkage study using primary care data found prevalence and incidence of schizophrenia was higher in more deprived areas [16]. A review similarly reported higher risk of psychotic disorders in deprived areas although adjustment for individual factors  (age, sex, ethnicity and social class) attenuated this association [48]. The assessment of deprivation in this study included income, employment, health, education, access to services, housing, community safety and physical environment [49]. Bhavsar et al. [50] demonstrated in highly urban areas, an association between area-level deprivation and schizophrenia incidence, was largely explained by age, gender, ethnicity, population density, high crime and low education. Whilst low income, poor housing and living environment did not independently predict incidence [50]. Therefore, the association between area-level deprivation and schizophrenia could be driven, partly, by specific social and urban contextual factors rather than deprivation alone. This research highlights the complexity of the relationship between deprivation and psychotic disorders.

Evidence shows schizophrenia prevalence is strongly socially patterned, since prevalence is higher in more deprived areas of society [51]. One possible explanation for this is that deteriorating mental state results in lower social position (social drift) [51]. However, evidence has not found people with diagnosed schizophrenia move to more deprived or urban areas [16]. Another explanation is that social position is a contributing factor for mental illness (social causation) [51]. However, Sariaslan et al [52]. found genetic risk for schizophrenia predicts neighbourhood deprivation, which could be evidence against environmental causes for this association. Therefore, overall, individuals in deprived areas could be more at risk of mental disorders [13, 53] however the causation for this is unknown and likely complex.

Urbanicity has also been associated with increased schizophrenia risk [16, 17], particularly during adolescence [15], although findings are mixed internationally [18]. Increased risk of schizophrenia could be a result of elevated exposure to environmental stressors, including air pollution [54, 55]. Higher residential exposure to NO_2_, NO_x_, PM_2.5_ and PM_10_ has been associated with increased mental health service use and incidence of schizophrenia spectrum disorders [56, 57]. However, in our study, pollutant levels showed minimal variation between the general population and those diagnosed with schizophrenia and other psychotic disorders. This may reflect the geographical and demographic characteristics of Wales. Since, Wales is characterised by smaller, dispersed urban centres and substantial rural areas rather than large metropolitan environments. Hence, the deprivation-

### Strengths and Limitations

Few population-level studies have described the environmental contexts of individuals with severe mental disorders using linked routine health data. Despite, a growing body of evidence investigating the aetiology of ecological determinants and mental disorders [59], research and related interventions have largely focused on biological, psychological, and social determinants [60, 61]. This study contributes to addressing this gap; however, several methodological and data limitations should be acknowledged.

First, no further analyses beyond descriptive statistics were conducted. Therefore, the results are limited to identifying trends, as descriptive statistics cannot be used to establish causal relationships.

Second, air pollution exposure was assigned at the LSOA level (1,500 people approximately per LSOA) rather than at the residential address level. This introduces the potential for ecological fallacy, whereby all individuals within an LSOA are assigned the same air pollution value, despite known variation over short distances. However, increasing the spatial resolution increases the computational complexity of the analyses and was not feasible in the lifetime of this study. Measuring air pollution exposure at a more individual level is a common difficulty [62]. Since participants may attend multiple locations throughout the day, such as work or school. Mental health outcomes are likely influenced by chronic long-term exposure to air pollution [57, 63]. However, this study was intended as a pilot analysis to assess the feasibility of linking area-level air pollution exposure with mental health outcomes. Accordingly, only a single year of air quality data (2016) was used to provide a snapshot of exposure while limiting analytical complexity. Although multi-year modelled air pollution data are available via DEFRA, cumulative or long-term exposure assessment was beyond the scope of this initial investigation and is being addressed in ongoing work using SAIL data. This likely underestimates the potential impact of air pollution on severe mental disorders and represents a notable limitation.

Regarding mental health data, the use of individual-level GP records provided high-resolution data. However, the percentage of schizophrenia/OPD patients was smaller than expected (0.088% of the cohort). Reliance on GP data alone is a likely explanation for the relatively small number of recorded diagnoses. This may limit the generalisability of the findings. Moreover, individuals who have been diagnosed with schizophrenia/OPD could be more likely to move residence. Lee and colleagues [16] showed individuals with severe mental disorders such as schizophrenia were more likely to move compared to the general population. Therefore, in this study, the exclusion of individuals who moved residence could have contributed to excluding some cases of schizophrenia/OPD. Additionally, all individuals in the study were followed for the full three-year period, resulting in a survivor cohort. Consequently, suicide and premature mortality among people with schizophrenia/OPD may have influenced the size of the psychiatric cohort [64]. An additional limitation is that current diagnoses were not used. As previously justified, 1 July 2016 to 30 June 2019 was selected as the study period to avoid the COVID-19 pandemic (2020–2021). During this period, GP access was difficult and disrupted [26]. A thematic analysis of 10,089 people reported difficulties booking appointments, appointment’s not meeting people’s needs and a lack of access to regular treatment and medication [26]. Hence, this disruption is likely to have interfered with diagnostic recording and may have introduced bias.

The higher mean age of individuals at the study start date may also be explained by the restriction to individual who did not change LSOA in the 3-year period. Younger age groups, in which residential mobility is higher, particularly those aged 19–29, may therefore be underrepresented.

ONS data indicate that young adults are the most likely to move residence, with a peak at age 19, reflecting transitions into higher education [65].

urbanicity–pollution gradient observed in highly urbanised settings may be less pronounced [58].

### Implications for Future Research

Understanding the environmental contexts of individuals diagnosed with severe mental disorders is crucial for identifying potential risk factors, resilience mechanisms, and protective factors. This is particularly important because this population is at a higher risk of poor physical health and reduced life expectancy compared with the general population [7].

Future research should examine the environments in which people with schizophrenia/OPD reside and work. Factors that could be investigated include pollution levels, greenness, urban versus rural settings, and socioeconomic deprivation. Studies could aim to include a more representative range of diagnoses by collecting data from various mental health settings, including general practice, inpatient, and outpatient hospital records, ideally at the population level. The use of up-to-date records is recommended wherever possible.

Research would also benefit from improved exposure assessment methods that consider multiple daily locations, including home, work, and school, as well as indoor exposures, to more accurately capture individuals’ everyday environmental interactions. It is also recommended future work incorporates multi-year exposure data to better understand long-term environmental impacts on severe mental disorders.

## Conclusions

In this population-wide descriptive study, individuals with schizophrenia or other psychotic disorders were disproportionately represented in more deprived areas. Distributions across urban and rural settings, as well as area-level air pollution exposures, were broadly similar to those in the general population. These findings highlight the importance of socioeconomic context when investigating inequalities in severe mental illness. Linked environmental and healthcare datasets provide opportunities for future longitudinal research. Such studies could examine the relationships between deprivation, urbanicity, environmental exposures, and severe mental disorders.

## Supplementary Files

Supplementary Appendices

## Data Availability

The data used in this study was accessed through Secure Anonymised Information Linkage (SAIL) Databank. Access to SAIL requires approval from the IGRP. Approved researchers can access the data *via* a privacy-protecting safe haven and remote access system, termed SAIL Gateway. The project number was 1413.
