# Supplementary material for: Ascertaining Household-Level Exposure to Air Pollution: Socio-Demographic Patterning and Urban-Rural Differences in People with Schizophrenia and Other Psychotic Disorders and the General Population
Source: Int J Popul Data Sci. 2026 Jul 2;11(1):3360. doi: 10.23889/ijpds.v11i1.3360 (PMC13386640; doi:10.23889/ijpds.v11i1.3360)

Appendix A: Code used in SAIL

A.1 Installation

```
install.packages("sailr");
library(sailr);
install.packages("tidyverse")
library(tidyverse)

conn <- sail_open();

q <- "SELECT * FROM SAILW1413V.JL_COHORT";
```

A.2 Descriptive statistics for all participants

```
#Number of all participants
nrow(data)

#Male (1)
sum(data$gndr_cd==1)

#Female (2)
sum(data$gndr_cd==2)

#Min, Q1, Median, Mean, Q3, Max Age
summary(data$age)

#Environment
unique(data$urban_rural_inception)
#Rural and fringe
sum(data$urban_rural_inception=="Rural town and fringe")
sum(data$urban_rural_inception== "Rural village and dispersed in a sparse setting")
sum(data$urban_rural_inception=="Rural town and fringe in a sparse setting")
sum(data$urban_rural_inception=="Rural village and dispersed")
#City and town
sum(data$urban_rural_inception=="Urban city and town")
sum(data$urban_rural_inception=="Urban city and town in a sparse setting")

#Deprivation
unique(data$wimd2019_quintile_desc_inception)
sum(data$wimd2019_quintile_desc_inception=="1. Most deprived ")
sum(data$wimd2019_quintile_desc_inception=="2 ")
sum(data$wimd2019_quintile_desc_inception=="3 ")
sum(data$wimd2019_quintile_desc_inception=="1. Least deprived ")
```

A.3 Descriptive statistics of the schizophrenia and OPD cohort

```
#All
sum((data$opd==1||data$schiz==1),na.rm=T)

# People with only schiz
sum((data$schiz==1&data$opd!=1),na.rm=T)
# People with only OPD
sum((data$opd==1&data$schiz!=1),na.rm=T)
# People with both
sum((data$opd==1&data$schiz==1),na.rm=T)

#Sex
sum(((data$opd==1|data$schiz==1)&(data$gndr_cd==2)),na.rm=T)

#Min, Q1, Median, Mean, Q3, Max Age
summary(data$age, data$opd==1|data$schiz==1)

#Rural and fringe
sum((data$urban_rural_inception=="Rural town and fringe") &(data$opd==1|data$schiz==1),na.rm=T)
sum((data$urban_rural_inception== "Rural village and dispersed in a sparse setting")&(data$opd=
=1|data$schiz==1),na.rm=T)
```

```
sum((data$urban_rural_inception=="Rural town and fringe in a sparse setting")&(data$opd==1|data$
schiz==1),na.rm=T)
sum((data$urban_rural_inception=="Rural village and dispersed")&(data$opd==1|data$schiz==1),na.rm=T)

#City and town
Sum((data$urban_rural_inception=="Urban city and town")&(data$opd==1|data$schiz==1),na.rm=T)
sum((data$urban_rural_inception=="Urban city and town in a sparse setting")&(data$opd==1|data$schiz
==1),na.rm=T)

#Deprivation
sum((data$wimd2019_quintile_desc_inception=="1. Most deprived ")&(data$opd==1|data$schiz==1),na.rm=T)
sum((data$wimd2019_quintile_desc_inception=="2 ")&(data$opd==1|data$schiz==1),na.rm=T)
sum((data$wimd2019_quintile_desc_inception=="3 ")&(data$opd==1|data$schiz==1),na.rm=T)
sum((data$wimd2019_quintile_desc_inception=="1. Least deprived ")&(data$opd==1|data$schiz==1),na.rm=T)
```

A.4 Air pollution values

```
#Air pollutants for All Participants
summary(data$pm10_mean)
summary(data$pm25_mean)
summary(data$nox_mean)

#Air pollutants Schizophrenia and Other Psychotic disorders Cohort
summary(subset(data, data$opd==1|data$schiz==1))
```

A.5 Code used to create Boxplots and histograms

```
#Type of distribution
#PM10
boxplot(data$pm10_mean)
hist(data$pm10_mean)

#PM2.5
boxplot(data$pm25_mean)
hist(data$pm25_mean)

#NOx
boxplot(data$nox_mean)
hist(data$nox_mean)
```

Appendix B: Distribution of the variables

Figure B.1: Boxplots and histogram of the NO_x (A and B), $PM_{2.5}$ (C and D), and PM_{10} (E and F) means

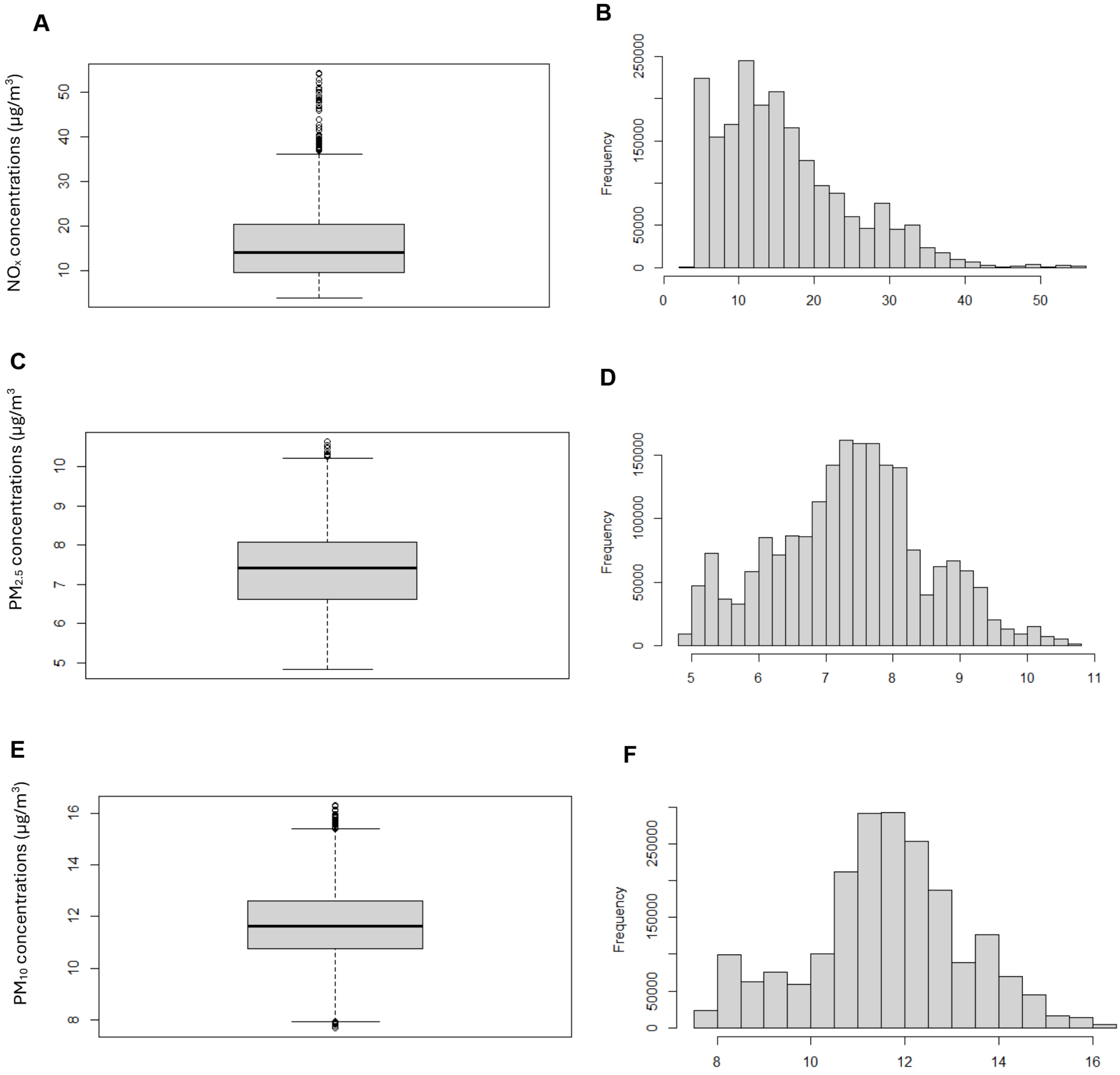

Supplement: Supplementary Appendices [file ijpds-11-3360-s001.pdf]
